# Enhanced resolution and sensitivity acoustic-resolution photoacoustic microscopy with semi/unsupervised GANs

**DOI:** 10.1038/s41598-023-40583-x

**Published:** 2023-08-17

**Authors:** Thanh Dat Le, Jung-Joon Min, Changho Lee

**Affiliations:** 1 Department of Artificial Intelligence Convergence, Chonnam National University, Gwangju, 61186 Korea; 2 Department of Nuclear Medicine, Chonnam National University Medical School and Hwasun Hospital, 264, Seoyang-ro, Hwasun-eup, Hwasun-gun, 58128 Jeollanam-do Korea

**Keywords:** Engineering, Optics and photonics

## Abstract

Acoustic-resolution photoacoustic microscopy (AR-PAM) enables visualization of biological tissues at depths of several millimeters with superior optical absorption contrast. However, the lateral resolution and sensitivity of AR-PAM are generally lower than those of optical-resolution PAM (OR-PAM) owing to the intrinsic physical acoustic focusing mechanism. Here, we demonstrate a computational strategy with two generative adversarial networks (GANs) to perform semi/unsupervised reconstruction with high resolution and sensitivity in AR-PAM by maintaining its imaging capability at enhanced depths. The b-scan PAM images were prepared as paired (for semi-supervised conditional GAN) and unpaired (for unsupervised CycleGAN) groups for label-free reconstructed AR-PAM b-scan image generation and training. The semi/unsupervised GANs successfully improved resolution and sensitivity in a phantom and i*n vivo* mouse ear test with ground truth. We also confirmed that GANs could enhance resolution and sensitivity of deep tissues without the ground truth.

## Introduction

After the first discovered in 1880^1^, photoacoustic effect contributed to open a new promising bioimaging technology called photoacoustic imaging (PAI). Owing to the rapid development of high-performance lasers, advanced acoustic sensors, and powerful computing systems, the PAI technology can provide dual image contrast by merging the strengths of optical and acoustic imaging. When a nanosecond pulsed laser is illuminated on a target sample, the biological molecules absorb the specific laser energy and generate broadband acoustic waves via thermoelastic expansion. These emitted acoustic waves are acquired using acoustic transducers, and 2D/3D images are generated through reconstruction algorithms. PAI enables maintenance of ultrasonic resolution even through deep biological tissues owing to the relatively low scattering of acoustic wave^2–4^. Based on molecular absorption at specific wavelengths, PAI provides information on structures, such as vascular network, lipids, and collagen^5–8^, in addition to functional information, such as concentration of hemoglobin, oxygen saturation, blood flow rate, and metabolism rate^9–15^. The superficiality of PAI thus contributes to advancement from basic science to clinical translation^16–18^.

Additionally, PAI can provide multiscale images selectively based on laser focusing and acoustic capture configuration. PAI systems are mainly of two types: photoacoustic tomography (PAT) and photoacoustic microscopy (PAM). PAT enables deep-tissue imaging with multielement transducers and a broad laser beam illumination for enhanced real-time acquisition speed without mechanical scanning approaches^4, 5^. Contrarily, PAM provides superior lateral resolution and sensitivity in shallow areas using a focused laser or acoustic beam along with fast lasing and scanning techniques^6^.

PAM is often categorized by designing a tightly focused laser beam to achieve high lateral resolution called optical-resolution PAM (OR-PAM), which produces sharp lateral resolution and reconstruction of time-resolved photoacoustic signals from up to 1 mm depth^19–21^. Based on the relatively less focused acoustic beam, PAM provides enhanced depth penetration via acoustic-resolution PAM (AR-PAM) for depths over 1 mm and up to several millimeters. Unfortunately, lateral resolution with the acoustic focusing approach is relatively poorer than that of OR-PAM because of the intrinsic acoustic resolution limitation^6, 7^.

To enhance the image quality and resolution of AR-PAM while maintaining its deep imaging capability, Tang et al. proposed a dual-focused synthetic aperture focusing technique (DF-SAFT) for AR-PAM that improved the signal intensity by two times, but the performance was highly dependent on fabrication skill^22^. E. Vienneau et al. reported a dual orthogonal ultrasonic transducer that allowed dual-view signals^23^ in nonlinear operations, which could affect quantitative measurements of the concentrations of molecules, such as blood oxygenation. M. Mozaffarzadeh et al. applied the double-stage delay-multiply-and-sum (DS-DMAS) algorithm to conventional AR-PAM combined with SAFT^24^, which incurred a higher computational complexity. Another popular solution was to use signal filtering techniques, such as FIR filtering^25^, empirical mode decomposition (EMD)^26^, and wavelet-based^27^ filtering to enhance acoustic signals or integration with OR-PAM systems to show the overlay of blood vessels (captured by AR-PAM) and smaller microvessels (captured by OR-PAM) with well-quality lateral resolution while maintaining imaging depth^28^. However, the signal filtering techniques used in AR-PAM scanning require repeated recording from the same position or the use of Fourier transform that slows the speed of real-time monitoring. Integrating the OR-PAM configuration has shown that there is no actual enhancement of the resolution.

Because of these limitations, deep-learning methods that generally enhance PAI performance are regarded as new solutions^29–31^, such as enhancing contrast with reconstruction under low-illumination laser^32–34^, removing artifacts with recovery of the out-of-focus regions^35–38^, and super-resolution transformation^39^. J. Kim et al. introduced a 3D U-Net to reconstruct high-density super-resolution images from fewer 3D label-free localizations of OR-PAM^40^, but this approach required downscaling to sparse localization because of limitations with the GPU memory. Another research, Z. Zhang et al. presented a deep CNN network that provided model-based iterative enhancement algorithms to recover the latent clean image from its degraded counterparts^41,42^. Most studies use convolutional neural networks (CNNs) that learn to minimize the Euclidean distances between the predicted and ground-truth properties (U-Net, AlexNet, ResNet, etc.), but decision-making with CNNs requires expert knowledge^43^. To distinguish classification of the output image while simultaneously training a generative CNN to minimize loss, the recently proposed generative adversarial networks (GANs) can solve the generative modelling problem^44–46^, which specifically designed for synthesize new data instances that resemble a given dataset. In particular, several researchers have tried to use GANs: S. Cheng et al. introduced an approach with Wasserstein GAN and gradient penalty (WGAN-GP) as the training network to transform low-resolution AR-PAM images to match high-resolution OR-PAM images obtained at the same depth^47^. Unfortunately, only the 2D maximum amplitude projection (MAP) images were processed here, which limits the accuracy of the depth area information. Y. Zhou et al. reported application of a conditional adversarial network (cGAN) to suppress the side lobes and enhance the depth of focus of a Bessel beam from a Gaussian beam^48^. However, these reported models required manual pairing, which causes difficulties.

In this study, we used the dual AR/OR-PAM system for data mining with a customized agar phantom embedded with three hairs and in vivo mouse ear with manual labeling for conditional GAN (cGAN) and automatic labeling without paired samples for the cycle-consistent GAN (CycleGAN). These two-representative semi/unsupervised GANs were compared for their performance enhancing abilities of low-resolution AR-PAM b-scan images to match the high-resolution OR-PAM b-scan images by measurement of the sensitivity parameters and differences in lateral/axial profile similarities. We further applied these GANs to a deep AR-PAM system to demonstrate quality enhancement with better lateral resolution at a greater depth without the validation from the OR-PAM configuration.

## Results

### Phantom study and quantitative evaluation

**Figure 1:**
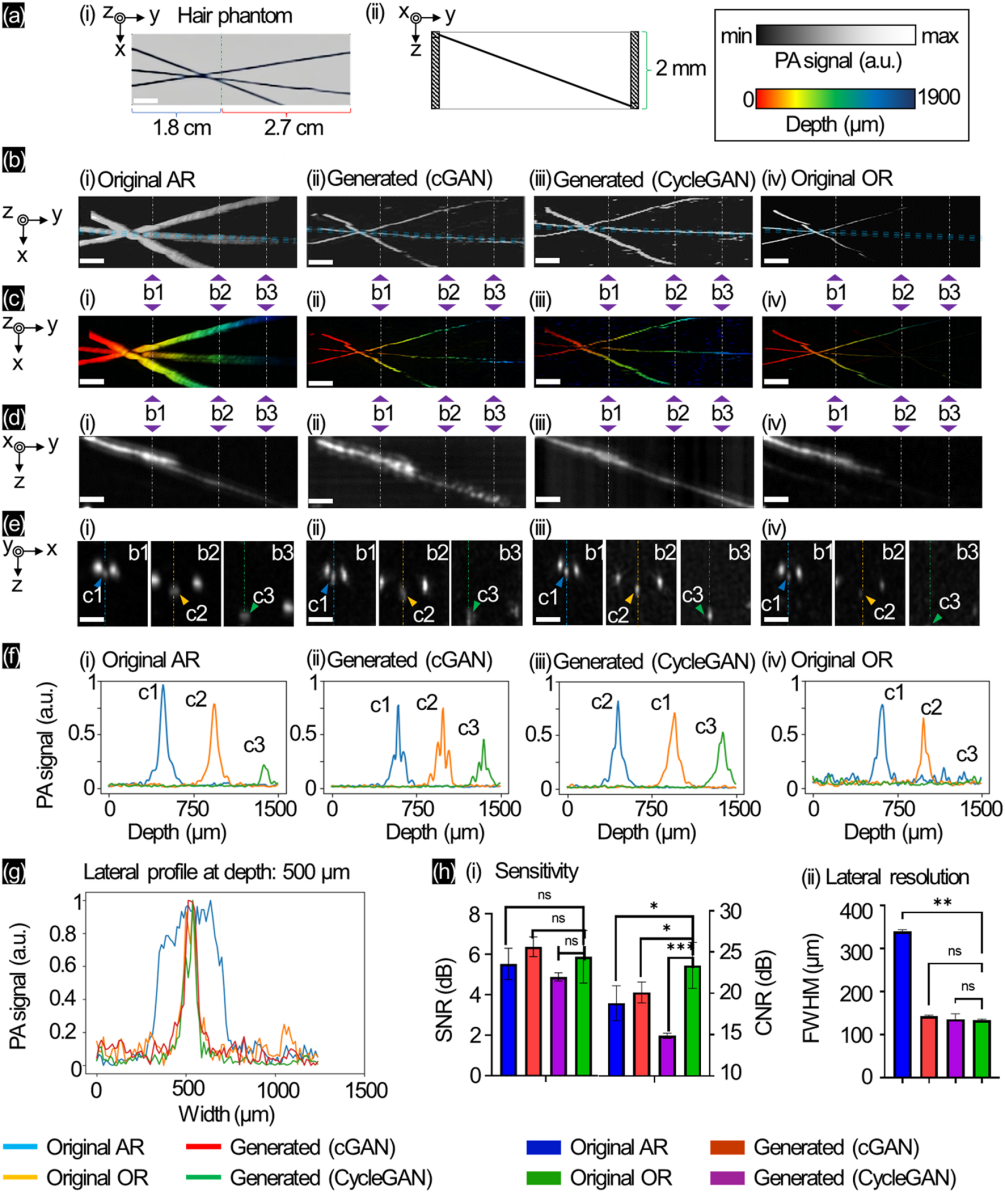
AR/OR-PAM images and generated OR-PAM images with GANs for different depths of the three-hair phantom. (**a**) Photograph of the hair phantom, (**b**) MAP images, (**c**) Corresponding depth-resolved MAP images with (**d**,**e**) Cross-sectional images of the middle hair along x–y plane and markers (b1–b3) along x–z plane, respectively. (**f**) The measured axial profiles at markers in (**e**) (i.e., c1: blue—c2: orange—c3: green), (**g**) Lateral profile comparison at 500 μm depth (yellow-diamond marked on (**e**)), (**h**) Sensitivity details with lateral resolution of the hair phantom. Scale bar: 500 µm (*p-value: (***) < 0.01, (**) < 0.05, (*) < 0.1, (ns) > 0.1).

To demonstrate enhanced depth imaging with the proposed GANs and image quality improvement in a well-prepared phantom (Fig. 1a), we used AR- and OR-PAM modes to find the maximum imaging depth achieved by both systems. The illustration (right) in Fig. 1a shows the phantom containing three human hairs at different depths from the top surface (left) to 2 mm depth (right). In Fig. 1b, we present the MAPs of the original AR / generation by cGAN / generation by CycleGAN / original OR. The series shows the depth mapping (Fig. 1c) with the central hair cross-sectional image along the y–z plane (Fig. 1d). Three cross-sectional images along the x–z plane (Fig. 1e) named “b1”, “b2”, and “b3” are selected to export the axial profiles (Fig. 1f) of the center hair that clearly shows the differences among AR-PAM mode at the deepest part of the hair (up to 1.9 mm) in Fig. 1e(i). The OR-PAM mode only shows the hair at ~ 1 mm (Fig. 1d(iv),e(iv)), where the deeper part of the hair cannot be easily recognized because the signal at 1.9 mm depth is low (same as background). Applying both cGAN (Fig. 1d(ii),e(ii)) and CycleGAN (Fig. 1d(iii),e(iii)), we identified the hair at a greater depth than the original AR. To prove this, we also acquired the lateral profile of the hair signal at 500 μm depth, which showed the smaller diameters of both the original OR and generated OR (~ 150 μm) from the original AR (~ 700 μm), as shown in Fig. 1g. Quantitatively, in Fig. 1h, we measured the sensitivity by calculating the signal-to-noise ratio (SNR) and the contrast-to-noise ratio (CNR) from the MAP images. For SNR, the signal between the original OR (5.8 ± 1.7 dB) and original AR (5.5 ± 0.8 dB) had no significant difference (p-value > 0.1). Therefore, neither trained network generated enhanced signals with cGAN (6.4 ± 0.3 dB) and CycleGAN (4.9 ± 0.1 dB). The CNR of original OR showed the highest value (23.4 ± 3.1 dB), converse to that of the original AR (18.8 ± 2.6 dB). The measured contrast was observed in the generators of both cGAN (20.1 ± 1.7 dB) and CycleGAN (14.9 ± 0.5 dB). To estimating sensitivity at difference depth from 100 to 1600 µm, we measured the SNR at different 3 depth positions as shown in Supplementary Fig. S1a. At the position R3, although the original OR showed nothing (0.023 ± 0.015 dB) and the original AR showed a low signal (0.2 ± 0.1 dB), PA signals at the same position were recovered well by cGAN (3.1 ± 0.3 dB) and CycleGAN (4.4 ± 0.1 dB). By measuring the full width at half maximum (FWHM) of the hair diameter, the original AR mode showed a larger diameter of the hair (338 ± 7 μm) than the original OR mode (134 ± 5 μm). Using trained GANs, the FWHM of the original AR was enhanced by both cGAN (144 ± 12 μm) and CycleGAN (136 ± 20 μm). In short, this hair phantom experiment showed the deeper parts from the AR-mode with better FWHM (~ 80%) than the OR-mode, and the recovery using GANs was successful for enhancing imaging resolution. To check its lateral resolution variance by imaging depth changing, we also measured the FWHM value at 6 different depth positions from 100 to 1100 μm as shown in Supplementary Fig. S2. The original AR and original OR showed at d3 (500 ± 10 μm) with 338 ± 7 μm and 134 ± 5 μm, respectively. Both cGAN and CycleGAN could maintain their FWHM well with 144 ± 12 μm and 136 ± 20 μm at d4 (700 ± 10 μm), respectively. Especially, CycleGAN achieved its latera resolution ability at d5 (900 μm) with 170 ± 38 μm. The structural similarity index measure (SSIM) value of the b-scans between the generated cGAN and original OR was 0.97 and that between the generated CycleGAN and original OR was 0.94. For more general visualization, we added the movie that showed 3D movies of AR-PAM and OR-PAM, and the recovering process of AR-PAM by cGAN and CycleGAN (Supplementary Fig. S3a and Movie S1).

### Quantitative evaluation of in vivo mouse ear experiments (w/ ground-truth)

**Figure 2:**
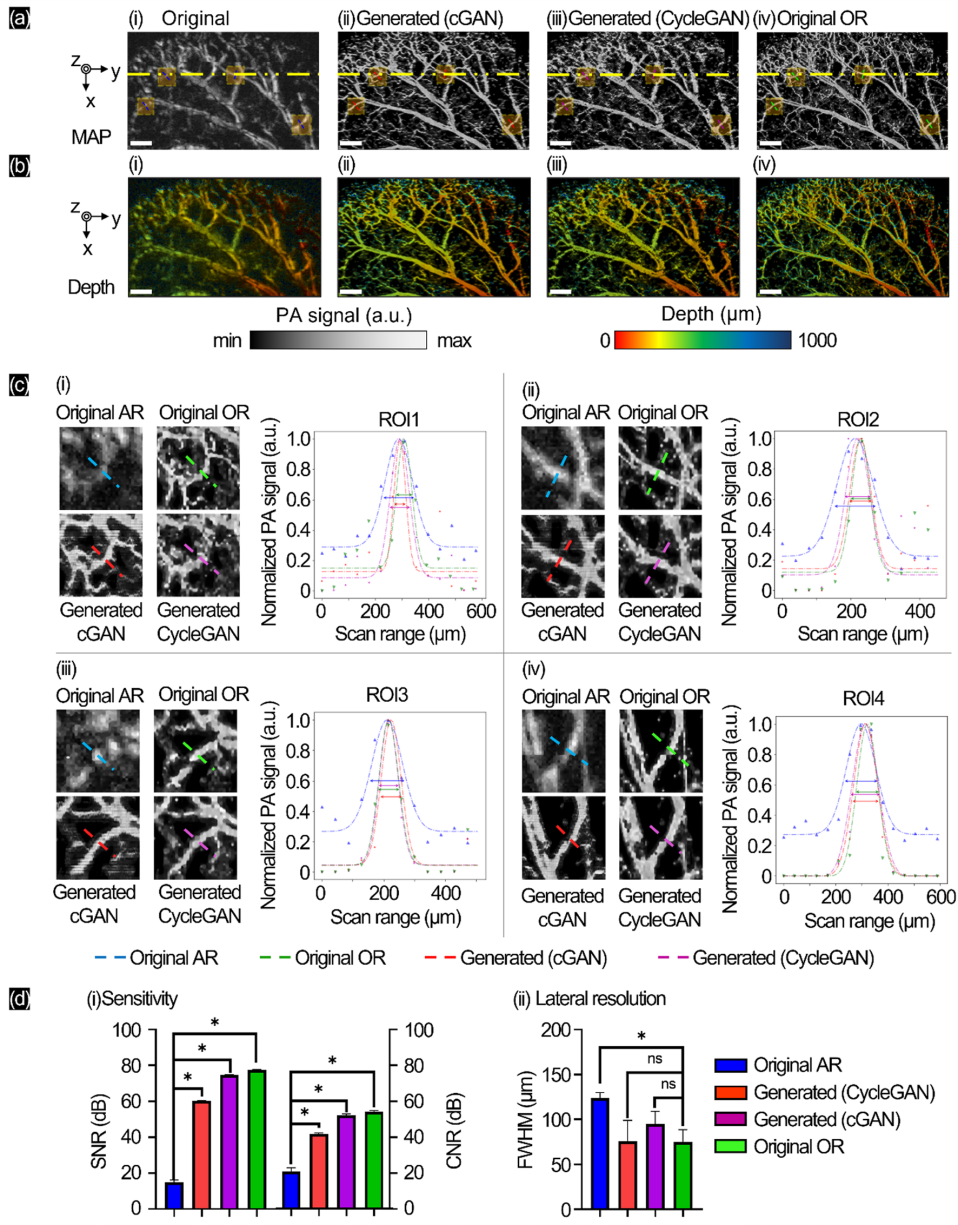
In vivo experiment on mouse ear with AR/OR-PAM images and generated OR-PAM images with GANs. (**a**) MAP images, (**b**) corresponding depth-resolved MAP images, (**c**) four different ROI MAPs in c(i–iv) and lateral profile comparisons of the original AR (blue), original OR (green), generated cGAN (red), and generated CycleGAN (purple). (**d**) Sensitivity and lateral resolution of mouse ear mapping. Scale bar 500 µm (*p-value: (*) < 0.1, (ns) > 0.1).

To demonstrate the in vivo experiment, we treated a mouse following the process described in the method section. We used AR- and OR-PAM modes in turn to quickly scan the mouse ear under anesthesia. In Fig. 2a, we present the MAPs of the original AR, generation by cGAN, generation by CycleGAN, and original OR with the same configuration as the previous experiment. The series shows the depth mapping (Fig. 2b). We specially marked four regions of interest (ROIs) (yellow rectangle marked in Fig. 2a and magnified in Fig. 2c). The lateral profiles of the original AR (blue) / original OR (green) / generated cGAN (red)/generated CycleGAN (purple) were cropped and plotted after Gaussian fitting. The diameter in the lateral profile based on FWHM is shown in the comparison. In Fig. 2d(i), we measured the sensitivity by calculating SNR and CNR. For SNR, the signal between the original OR (77.4 ± 0.4 dB) and original AR (14.9 ± 1.1 dB) showed large gaps in performance (original OR higher than original AR by ~ 519%). Therefore, both trained GANs generated enhanced signals, with cGAN (60.3 ± 0.1 dB) and CycleGAN (74.7 ± 0.1 dB). The CNR of the original OR was higher (53.9 ± 0.7 dB) than that of the original AR (19.6 ± 1.9 dB). The measured contrast in the cGAN generator was 20.2 ± 0.5 dB, which is worse than that of CycleGAN (27.6 ± 0.8 dB). We measured the lateral resolution by the vascular diameter (Fig. 2d(ii)), and the original AR mode showed a larger diameter (124 ± 5 μm) than the original OR mode (75 ± 12 μm). Using trained GANs, the lateral resolution of the original AR was enhanced by cGAN (76 ± 20 μm) better than CycleGAN (95 ± 12 μm). The SSIM value in MAP between the generated cGAN and original OR was 0.75 ± 0.02 and that between the generated CycleGAN and original OR was 0.74 ± 0.02. For more general visualization, we added the movie that showed 3D movies of AR-PAM and OR-PAM, and the recovering process of AR-PAM by cGAN and CycleGAN (Supplementary Fig. S3b and Movie S2), with B-scan profile at yellow line in Fig. 2a (Supplementary Fig. S4).

### Quantitative evaluation of in vivo deep mouse-back experiment (w/o ground-truth)

**Figure 3:**
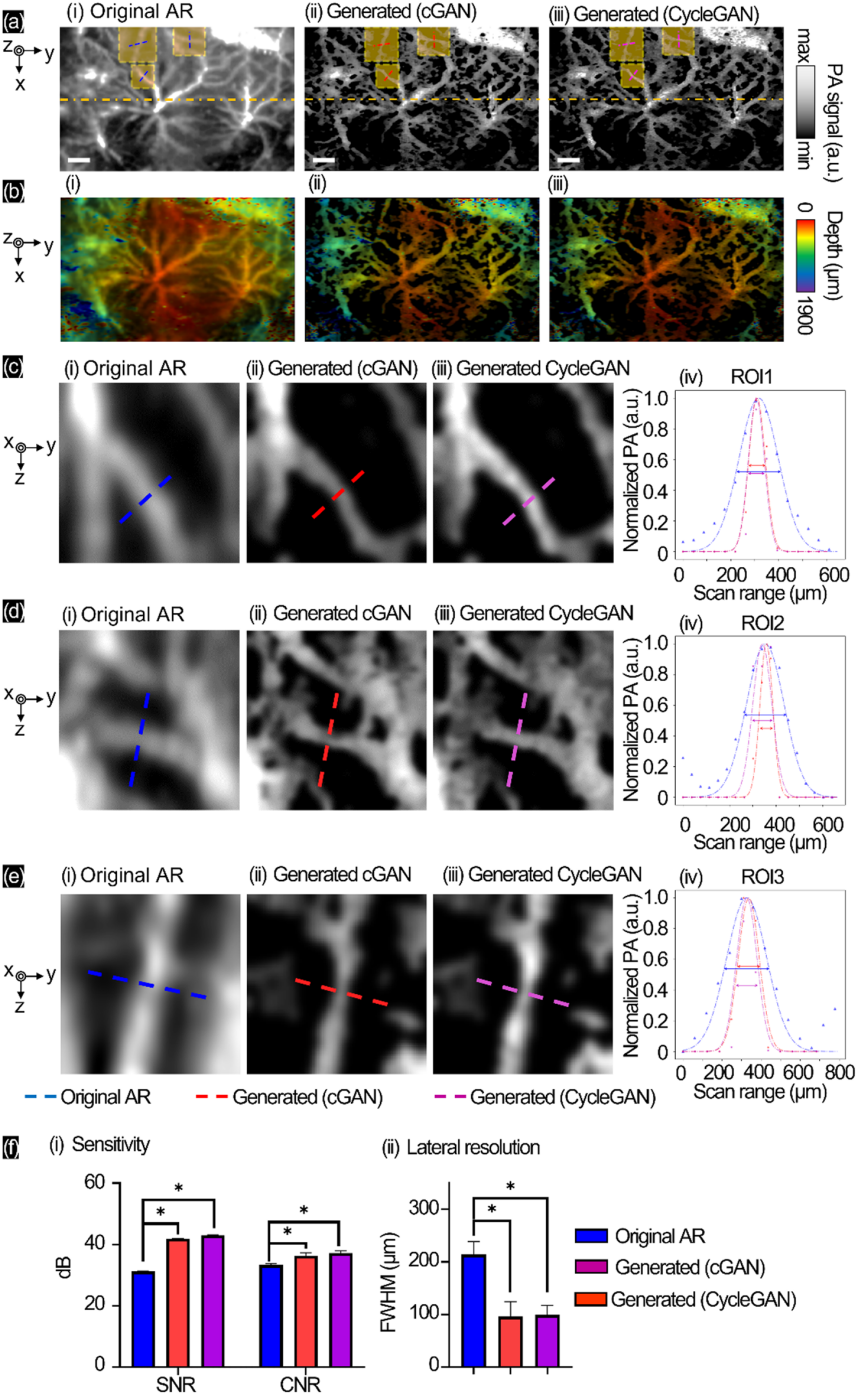
In vivo experiment of the side-back skin of mouse with deep AR-PAM imaging and generated OR-PAM images with GANs. (**a**) MAP images, (**b**) corresponding depth-resolved MAP images, (**c**–**e**) lateral profile comparisons of the original AR (blue), generated cGAN (red), and generated CycleGAN (purple). (**f**) Sensitivity and lateral resolution of mouse vascular mapping. Scale bar 1000 µm (*p-value: (*) < 0.1).

By applying the deep AR-PAM system alone, we conducted the same experimental process without the ground truth. We used the deep AR-PAM system to scan a side of the mouse’s back skin under anesthesia. In Fig. 3a, we present the MAP of the original AR, generation by cGAN, and generation by CycleGAN without original OR. The series shows the depth mapping (Fig. 3b). By choosing three ROIs (in Fig. 3c–e), the lateral profile of the original AR (blue, Fig. 3c(i),d(i),e(i)/generated cGAN (red, Fig. 3c(ii),d(ii),e(ii)/generated CycleGAN (purple, Fig. 3c(iii),d(iii),e(iii) were cropped and plotted after Gaussian fitting in Fig. 3c(iv),d(iv),e(iv). In Fig. 3f(i), we measure the sensitivity by calculating SNR and CNR from the MAP images. For SNR, the signal of the original AR (31.2 ± 0.1 dB) showed differences for both trained GANs, with enhanced signals for cGAN (41.9 ± 0.1 dB) and CycleGAN (43.0 ± 0.1 dB). The CNR of the original AR (33.3 ± 0.3 dB) was smaller than the measured contrast with the cGAN (36.3 ± 0.9 dB) and CycleGAN (39.2 ± 0.6 dB). We measured the lateral resolutions at the same position (Fig. 3f(ii)), and the original AR mode showed a vascular diameter of 218 ± 21 μm. Using the trained GANs, the lateral resolution of the original AR was enhanced by cGAN (95.7 ± 2.4 μm) better than CycleGAN (99.1 ± 1.6 μm). Finally, we presented the 3D movie of original AR scanning with the recovering process by cGAN and CycleGAN in Supplementary Fig. S3c and Movie S3, with quantitative measurement in different depth position in Supplementary Fig. S5. By specific ROIs at different cross-section depth areas, the top layer (d1, Supplementary Fig. S5) showed the highest PA signal level and could be used recovery as well with detail small vascular from original AR-PAM (SNR = 20.256 dB, CNR = 27.315 dB) to synthetic cGAN image (SNR = 37.742 dB, CNR = 32.871 dB) and synthetic CycleGAN (SNR = 42.809 dB, CNR = 26.963 dB). In deeper position (d2, Supplementary Fig. S5), the original AR-PAM (SNR = 26.124 dB, CNR = 31.507 dB) could show contrastive PA but lower signal level than surface, so the synthetic images could not recognize well (cGAN: SNR = 26.364 dB, CNR = 20.157 dB) (CycleGAN: SNR = 28.405 dB, CNR = 22.661 dB).

## Discussion

By demonstrating the proposed unsupervised and semi-supervised GANs for enhanced depth and image quality improvement in a customized phantom (Fig. 1a), we proved that the enhanced depth signal with the AR-PAM system was better than that with OR-PAM under 1 mm. The proposed GANs enhanced the source AR-PAM b-scan images and showed lateral resolutions similar to the OR-PAM b-scan images on the surface through FWHM measurements, as shown in Fig. 1g (close to ~ 80%). From the series showing the depth mappings (Fig. 1c) for the central hair cross-sectional image at the y–z plane (Fig. 1d), we chose three b-scan images at “b1”, “b2”, and “b3” to prove that both cGAN and CycleGAN work well. In conjunction with Supplementary Fig. S1, we also observed that both cGAN and CycleGAN were successful in effectively restoring the high PA signal in regions located at depths beyond 1400 µm when compared to the original AR and the original OR images. The measured lateral profiles of FWHM in both cGAN and CycleGAN could keep and recover well the original OR lateral resolution when original AR out-of-focus deeper than 700 µm (both GANs model are shallower 4 times than original AR), as shown in Supplementary Fig. S2. Despite the attenuation of the PA signal with imaging depth, the FWHM of the axial profiles in Fig. 1f remains relatively similar. The generated axial profile’s FWHM exhibits approximately 10% lower values compared to the originals. This can be attributed to the utilization of the same measurement transducer, resulting in comparable axial resolution. The quality measurements via SNR and CNR showed insignificant differences between the original OR and original AR (not significant), and we consider that both cGAN and CycleGAN did not improve SNR in the phantom experiment.

From the in vivo experiment with mouse ear monitoring by the dual AR/OR-PAM system, as shown in Fig. 2a, we present the MAP of the original AR-PAM with more blurring and loss of most of the small vascular area than the original OR-PAM. By applying the proposed GAN pretrained datasets, the generations by cGAN and CycleGAN could recover the missing vessels from the original AR b-scan images but not perfectly. The salt and pepper noise from the original OR caused unequal tissue distribution (skin, fat, blood, muscle, cartilage, and small hairs on mouse ear) as it was difficult to prepare a noise-free training dataset. The small gaps between the vasculature were hard to observe in the proposed GANs. In the specially marked four ROIs in Fig. 2c, the lateral profile of the original AR (blue) was significantly larger in diameter than the original OR (green), so the proposed generated cGAN (red) and generated CycleGAN (purple) successfully enhanced and recovered the unlined small vascular structures. Therefore, both trained GANs generated enhanced signals, with cGAN and CycleGAN having three times higher results than the original AR. For visualization, we showed the B-scan image (yellow line in Fig. 2a) and their lateral profile in Supplementary Fig. S4. The CNR of CycleGAN was higher by a few points (~ 2.9 dB) than both the cGAN generator, and no significant difference between original AR the original OR. In Fig. 2d(ii), we cropped the lateral profile to show single small vascular structures, where the proposed GANs converted the original AR to sharp images better by cGAN than CycleGAN. This in vivo experiment proved that better lateral resolution could be repeatedly recovered using GANs with similar structures as the original OR (~ 0.75 by SSIM). Even with unstable vessel structures, the sensitivity was successfully improved from the low-level original AR. The absorption properties in the custom human hair phantom (having the most amount of melanin) are stronger than those of soft biomaterials (blood, vessel borders, skin, etc.)^49^. These indicate that the GANs recovered complex patterns with blurring and noise under the skin and produced good effects by increasing the SNR.

To improve the process without the ground truth images, we used the deep AR-PAM system without OR-PAM to scan a mouse’s side back that showed the twisted vascular structures and changes based on depth. Here, the proposed GANs still produced better reconstruction and lateral resolution than the original AR (~ 190% sharper). We measured the lateral profiles at three ROIs (in Fig. 3c–e), which showed similar trends of sharp vascular structures and diameter from both the pretrained cGAN and CycleGAN. The measured sensitivity by SNR and CNR (Fig. 3f(i)) showed significantly different when compared with the original AR (decrease ~ 34% noise and ~ 18% enhancing contrast for the proposed GANs) (p < 0.1). We also checked the sensitivity at specific ROIs of different depth images as shown in Supplementary Fig. S5. The great visualization that showed the well recovery from saturating PA signal from near surface AR-PAM image (enhancing contrast and reducing noise) but be poorer at lower depth it showed unclear structures. Fortunately, the generated PAM images showed the detail vasculature map at more deep depth. Due to the use of different transducer specification in preparing the deep AR-PAM data for the testing dataset, both cGAN and CycleGAN models were unable to significantly enhance the lateral resolution as effectively as the OR-PAM system. Subsequent to the enhancement of the deep AR-PAM b-scan images, no thresholding or bandpass filter was employed. Nevertheless, notable contrast enhancement was observed in the synthetic b-scan images. That could be noticeable that thresholding and bandpass filter configuration in dual OR/AR-PAM was learned and applied by GANs models, so the manual denoising was able to pass temporarily.

In most experiments with GANs, researchers have only selected the best results they could show because the GANs are difficult to control while maintaining good conditions. To compare the differences between the cGAN and CycleGAN based on training time (epochs), we chose in vivo experiments that showed significant bias efficiency over the rest for the original OR (SNR, CNR, lateral resolution, and SSIM for p < 0.1 with the best weight). In Supplementary Fig. S6, we showed the epochs 10–50–100–150–200 as the marks and separately use cGAN and CycleGAN to generate results from the original AR in vivo dataset. The SNR (Supplementary Fig. S6a) shows balance during training time in both GANs. The CNR (Supplementary Fig. S6b) shows a similar value for cGAN as compared to the original AR (similar in the phantom experiment). Hence, the CNR was not improved by cGAN in both the phantom and in vivo experiments. However, the CycleGAN showed the highest score for 10 epochs and then decreased close to that of the original OR. This was opposite to the result of the phantom experiment, where the CNR of CycleGAN was lowest (~ 1.5 times less than original OR). The lateral resolution of cGAN was smaller than that of the original AR and had an earlier balance and similar diameter as the original OR. The generated signal by CycleGAN slowly decreased at 150 epochs and was close to that of the original OR. These observations were also proved by the SSIM via comparison of the original AR, generated cGAN, generated CycleGAN separately with the original OR as initial comparison (Supplementary Fig. S6d). Because the SNR and CNR values of the original AR were the smallest, and the lateral resolution was highest, the SSIM was also the smallest (0.14 compared with the original OR). The value generated by cGAN slowly increased (from ~ 0.68 to 0.8), following the same trend as the value generated by CycleGAN (from ~ 0.63 to 0.77), whose learning was best after 200 epochs. We conducted the custom hair phantom experiment with a simple condition (fixed diameter, simple structure) and the in vivo mouse ear experiment with a more complex condition (dynamic vascular structure based on time, flexible structure). These proved the efficiencies of both cGAN and CycleGAN with well-prepared samples. The problem with the mouse ear prepared dataset was that it did not contain multilayer information. However, enhancement for depth changes and noise-free lateral resolution are still efficient features of the deep AR-PAM scanning, as in the previous experiments with MAP^47^, b-scan image training^48^, or 3D training^40^. These results were verified by in/ex vivo animal experiments with random samples.

This study still has some major limitations. First, the model has a lot of parameters that should be carefully optimized cause the stability of the GAN is highly dependent on network architecture (our generator was based on ResNet-9 blocks, which can be modified to U-Net or another generator networks). Second, there were many factors that affected the choice of GPU (price was too high for the experiments, memory of GPU was not enough for full-scale b-scan, training time was too long to optimize the parameters, and training failure caused random corrupted memory during training). Lastly, there is a limitation with the dataset in that it does not cover all scales of biostructures (only proven with vascular shapes and not organs, lymph nodes, bones, etc.) and the dataset was prepared on the limited system specification with our own experiment configuration. To deal with current issues, we are going to design a translation standard which can combine our current GANs following different transducers configuration in simple GANs models. The GANs can be used to replace manual enhancing and reduce the processing time to be able for research and commercial PAM system.

## Materials and methods

### Dual AR/OR-PAM

**Figure 4:**
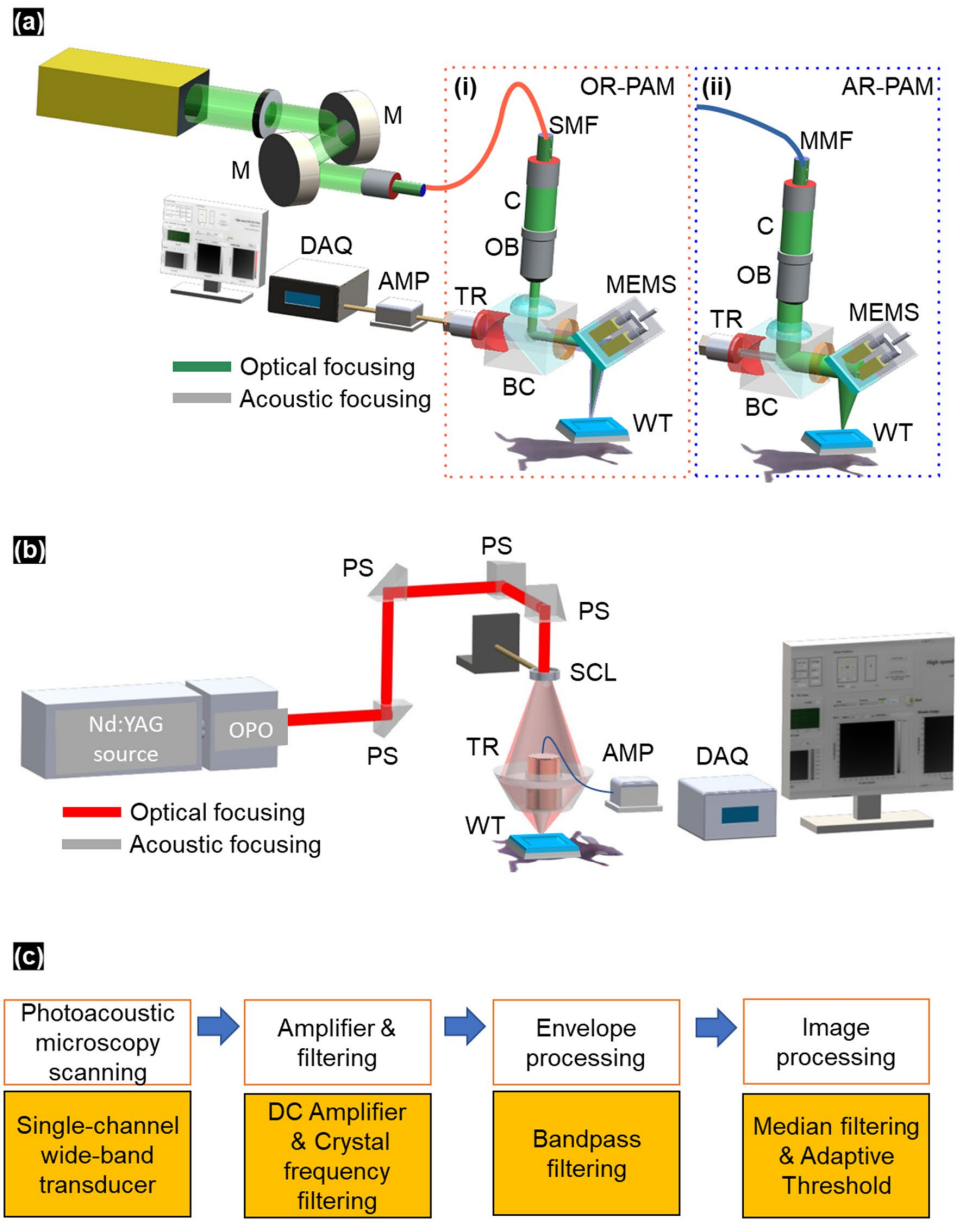
(**a**) System configuration of the dual AR/OR-PAM system: (i) Part of OR-PAM with optical focusing (green), (ii) Part of AR-PAM with acoustic focusing (gray), (**b**) system configuration of the deep AR-PAM system, (**c**) preprocessing workflow. *M* mirror, *SMF* single-mode fiber, *MMF* multimode fiber, *C* collimator, *OB* objective lens, *BC* beam combiner, *TR* transducer, *AMP* amplifier, *MEMS* MEMS scanner, *DAQ* data acquisition, *PS* prism, *SCL* conical lens, *OPO* optical parametric oscillator, *WT* water bath.

We prepared a dual-mode AR/OR-PAM system as shown in Fig. 4a. A pulsed laser with a central wavelength of 532 nm (SPOT-1010-200, 10 kHz repetition rate, Elforlight, France) served as the main source for the dual AR/OR-PAM system. The beam was first delivered to a collimator (C, F280APC-A, Thorlabs, USA) and separately connected to two optical fibers (i.e., single-mode fiber for OR-PAM and multimode fiber for AR-PAM) depending on the PAM operating mode. To achieve OR-PAM mode, a 5 μm single-core optical fiber (SMF, P1-405BPM-FC-1, Thorlabs, USA) was used, and the delivered beam was focused using a doublet lens (OB, AC254-060-A, Thorlabs, USA). To demonstrate AR-PAM mode, a 400 μm multicore optical fiber (MMF, M74L01, Thorlabs, USA) was used, where the focused beam was first passed through a homemade beam combiner (BC) coated by thin aluminum-sheet. The focused laser beam was then reflected and directed with a one-axis MEMS scanner (MEMS, OpitchoMS-001, 50 Hz, Opticho Inc., Ltd., Korea). The laser beam was absorbed by the biological tissue, and through subsequent thermal expansion, the acoustic signal was exposed to 360 degrees. The generated acoustic signal was reflected by the MEMS mirror and directed through the aluminum-prism to a high-frequency 50 MHz ultrasonic transducer (TR, V214-BC-RM, Olympus NDT, Japan). The whole transducer was aligned and immersed in the water bath. To amplify the acoustic signal, an amplifier (AMP, ZX60-3018G-S+, MiniCircuit, USA) was used, followed by crystal low-pass filtering (CLPFL-050, CRYTEK, USA). The analog signal was converted to digital signal with a high-speed digital board (DAQ, ATS9371, AlazarTech, Canada) that digitized the 10 μm depth-resolved analog signal to 16-bits digital A-scans before storage. To scan a large-field surface, two linear stepper-motorized stages (L-509-10SD00, Physik Instrumente, Germany) along the x–y plane and a MEMS scanner, with a 0.127 μm x-step and 0.386 μm y-step, were used to obtain data. The measured lateral resolutions in OR-PAM and AR-PAM were 12 μm and 85 μm, respectively. The axial resolution was 27 μm, which was fixed for the same transducer. For the synchronous control stages, a custom LabVIEW program was developed to manage the hybrid scanning system with a data acquisition board (PCIe-6321, NI Instruments, TX, USA). The laser was controlled at approximately 5 mJ/cm2 below the American National Standards Institute (ANSI) limit (20 mJ/cm2 under visible light). All data analyses and reconstructions were performed using MATLAB (R2020b, The MathWorks, MA, USA). The 3D visualization in Supplementary Fig. S3 was created by movie maker tools in 3D PHOVIS^50^.

### Deep AR-PAM

We configured the deep AR-PAM system^7^ as shown in Fig. 4b. A pulsed laser beam source with a central wavelength of 700 nm (Surelite OPO PLUS, Continuum, USA) was controlled by a Q-switched Nd:YAG laser (5 ns pulse duration and 10 Hz pulse repetition rate, SLII-10, Continuum, USA). The diverged ring-shaped beam configuration formed a spherical conical lens (AX2540-B, Thorlabs, USA) and a handmade optical condenser. Using this imaging setup, the dark-field light illumination was coaxially aligned with ultrasound focusing on water. The incident laser pulse energy was limited to below the ANSI safety limit of 20 mJ/cm^2^. The generated ultrasound waves were detected with a single-element 5 MHz ultrasonic transducer (V308, Olympus NDT, Japan) which immersed in the water bath. The axial and lateral directions were 150 μm and 590 μm, respectively.

### Data preprocessing

Figure 4c shows the data preprocessing flow chart. To prepare the dataset acquired with the dual AR/OR-PAM system, we saved all normalized b-scan images as stacks in a 3D volume array. The 3D volume was loaded as a single task during processing and separated into multiple frames with 256 × 256 pixels for each image. The random jitter function was disabled to avoid scaling errors. The photoacoustic amplitudes were normalized for the entire volume to ensure that the laser power remained identical. To reduce the background noise, we applied bandpass filtering based on difference ultrasound transducer properties to each envelope A-line dataset for dual AR/OR-PAM and deep AR-PAM system.

### Experimental sample preparation

As experimental materials, we prepared three samples: a custom-designed three hairs embedded phantom, a mouse ear, and a mouse back skin. These samples were prepared to monitor the sensitivity and structure of the objects and their effects on the training models.

A phantom embedded with three hairs was prepared to show different imaging depths and resolutions between the AR- and OR-PAM images. Using a 3D printer (da Vinci 1.0 Pro, XYZ printing Inc., USA)^51^, a white polylactic acid frame (30 × 50 mm) with three top and three bottom holes was designed to fix three long black hairs at linearly increasing depths from the surface to 5 mm. The frame was internally filled with 5 g of 1.5% concentration mold jelly (BD BACTO™ Agar, Becton–Dickinson and Company, USA)^52^ mixed with 3 g of milk and 100 mL pure water. We cooked this mixture at 80 °C for 5 min and stirred well until the Agar dissolved completely. A solid formed stably after 15 min in the refrigerator and maintained under a wet towel.

We prepared two in vivo samples for this study: a mouse ear for use with dual AR/OR-PAM scanning and a side-back skin of a mouse for deep AR-PAM scanning. Two healthy six-week-old male BALB/c mice (Orientbio, Korea) were used according to the instructions of laboratory animal protocols approved by the Institutional Animal care and Use Committee of Chonnam National University Hwasun Hospital (CNU IACUC-H-2020-35). The mice each weighed ~ 20 g and were anesthetized by intraperitoneal injection of a cocktail of Ketamine/Xylazine at 70/15 ratio. After removing the surface soft hairs, we placed the experimental mouse on a small animal table. For better acoustic coupling in vivo animal experiment, we used the ultrasound gel (hypo-allergenic water soluble, Sono Jelly, Meditop Co., Ltd, Korea). An isoflurane system (Luna Vaporiser, NorVap International Ltd., Barrowford, UK) was used for gaseous anesthetization, and a temperature table was used for short-term in vivo observations to maintain stable body condition of the mouse^53^. The energy of the laser pulse was ~ 5 mJ/cm^2^. Each sample was prepared and imaged separately. We scanned the mouse ear vasculature in both AR- and OR-PAM modes because the size of the mouse ear was small enough (10 × 10 mm^2^) for fast scanning. For the side-back skin of mouse scanning (100 × 50 mm^2^), the deep AR-PAM system required a longer scanning time (~ 20 min) owing to the mechanical scanning speed and laser repetition rate^7^. Hence, we divided the volume into four vertical stacks and displayed each b-scan image with 300 × 512 pixels to acquire the 3D volume.

### Image-to-image GANs

Typical GAN models have been used in various imaging tasks^54^. To clarify the opposing relationships between AR-PAM and OR-PAM b-scan images, we applied different pixelwise contrastive GAN models, cGAN and CycleGAN, to learn the mapping functions. This was to prove the effectiveness of unsupervised learning with different datasets (labeled in cGAN and label-free in CycleGAN) in AR-PAM for enhancement of the intended sample (phantom, mouse ear vascular with ground truth from OR-PAM scanning) and uncertain sample (mouse’s back skin without ground truth).

**Figure 5:**
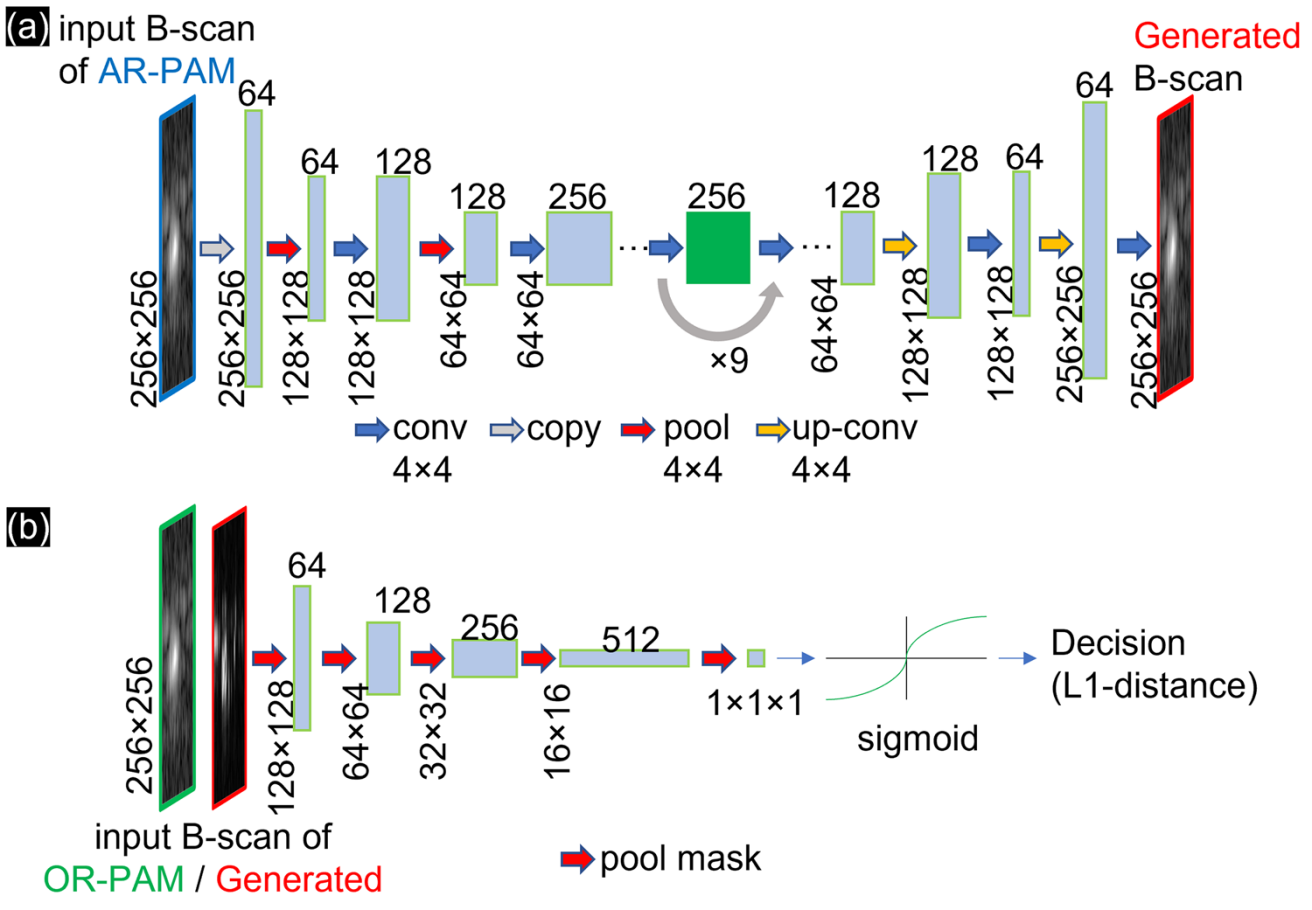
(**a**) Generator and (**b**) discriminator of the GANs.

The schematic of the GANs consists of competing between generators and discriminators to create and discern in the network. In both cGAN and CycleGAN, we used ResNet-9 blocks (Fig. 5a) in the generator to avoid overfitting and achieve lower training errors when the details increase in the b-scan images. The input to the generator was the 256 × 256 patches of b-scans from AR-PAM. Between the encoder and decoder layers, the generator used nine residual blocks (green block) containing the identify function as one of the elements. The output of the generator was 256 × 256 patches of b-scan images. The discriminator used was a Markovian discriminator (Fig. 5b) that relied on a term to force low-frequency accuracy even with large b-scans. By multiplying the generated patch b-scan images with the target patch OR-PAM b-scan images, the discriminator encoded multiple pool mask layers to a 1 × 1 decision vector by the sigmoid function. Finally, the L1 distance was updated loss distance in the generator.

To replicate a probability photoacoustic amplitude distribution, the GANs were subjected to get the minimax task (Eq. 1) between the generator and discriminator to discriminate the learning process. In our study, that minimax task performed distribution of the generated data (G) between the original/source AR-PAM and original/target OR-PAM data (O). This was aimed at distinguishing the actual b-scan image map (X, Z) with the discriminator (D). The final generator ${G}^{*}$ and discriminator ${D}^{*}$ were created using the minimum argument ($arg$) of loss function $L\left(G,D\right)$ to minimize the generator ${min}_{G}$ and maximize the discriminator ${max}_{D}$ to ensure lower difference.

1 $${G}^{*},{D}^{*}=\mathit{arg}\,{\mathit{min}}_{G}{\mathit{max}}_{D}L\left(G,D\right)$$

**Figure 6:**
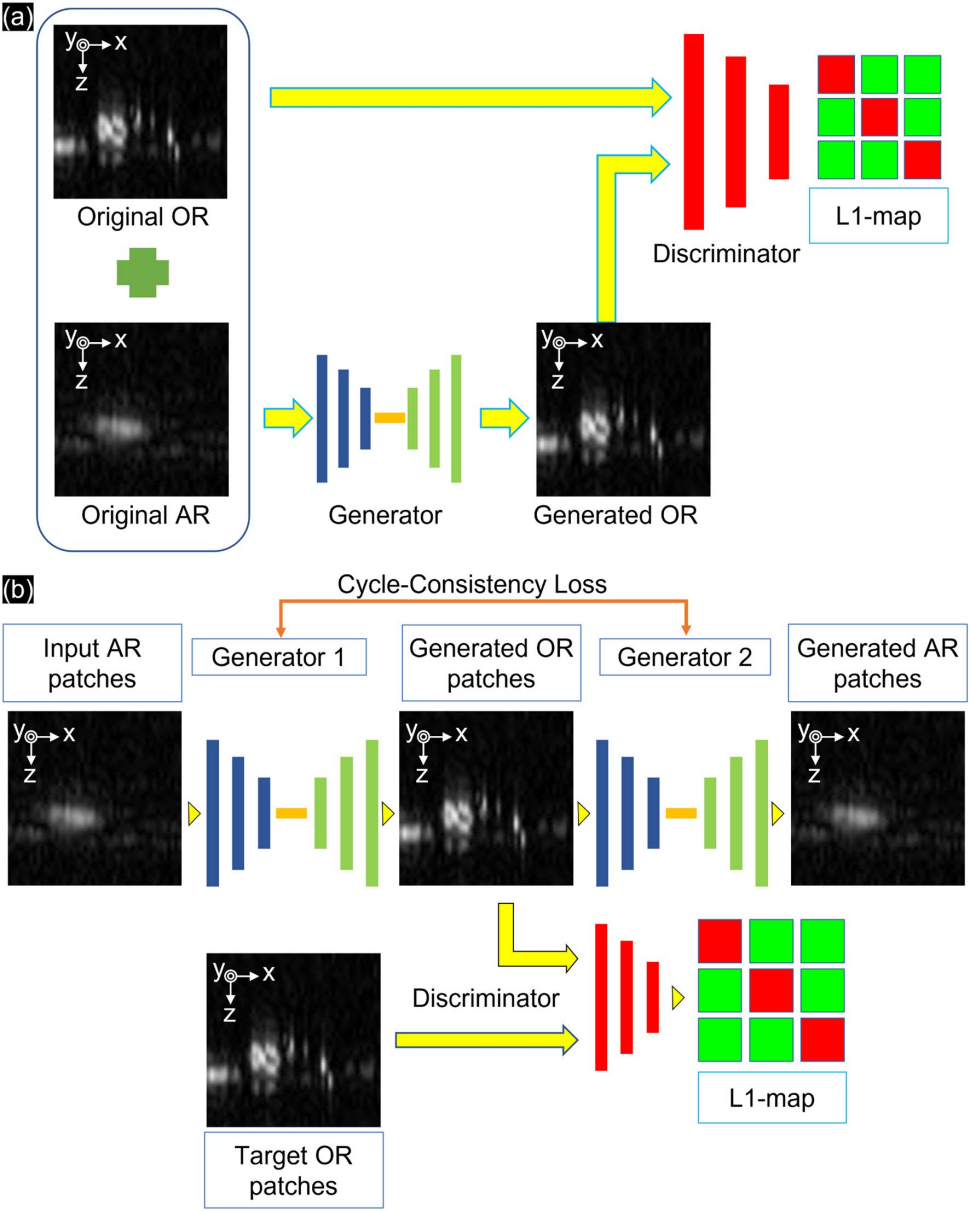
Extracting samples during 3D training with the (**a**) cGAN and (**b**) CycleGAN models.

P. Isola et al. proposed the modified cGAN^55^ to learn features from AR-PAM b-scan images paired with OR-PAM b-scan images. In Fig. 6a, the cGAN is shown with one generator and one discriminator. By labeling the AR-PAM b-scan images with the OR-PAM b-scan images in a one-to-one manner, the generator encodes the AR-PAM b-scan images to a 1D vector as “features”. The residual blocks bypass the nonlinear layers with an identity mapping to remove the AR-PAM’s features. The decoder part of the generator adds the “learned features” that are updated from the previous epoch. By comparing the generated b-scan images with the paired OR-PAM b-scan images, the discriminator then encodes the multiple generated/OR to a sigmoid activation function that creates the updated decision for the next epoch. Using the loss function (Eq. 2) for cGAN (${\mathcal{L}}_{cGAN}$), cGAN drives the generator ($G$) from the expected values ($E$) of the original AR b-scan images ($A$) and paired pixelwise discriminator ($D$) from the original OR b-scan images ($O$).

2 $${\mathcal{L}}_{cGAN}(A,O)={\rm E}_{A,O }\left[logD\left(A,O\right)\right]+{E}_{A}\left[\mathit{log}\left(1-D\left(A,G\left(A\right)\right)\right)\right]+{E}_{A,O}[{\Vert O-G(A)\Vert }_{1}]$$

Based on image-to-image translation, J. Y. Zhu et al. introduced the unpaired learning method using CycleGAN^56^. Inspired by the CycleGAN structure, we denoted the data distribution as two groups of PAM images with expected values of original AR-PAM b-scan images (${E}_{A}$) and original OR-PAM b-scan images (${E}_{O}$) without pairing. Similar to cGAN, the process with the generator/discriminator learned from the updated decision that affected the next epoch. In Fig. 6b, by identifying the backward-looking generator $(\overline{G })$, the reverse is confirmed so that can the cGAN can be used in both directions (from AR to OR—${\mathcal{L}}_{cGAN}\left(A,O\right)$ and inverse—${\mathcal{L}}_{cGAN}\left(O,A\right)$).

### Network performance

**Figure 7:**
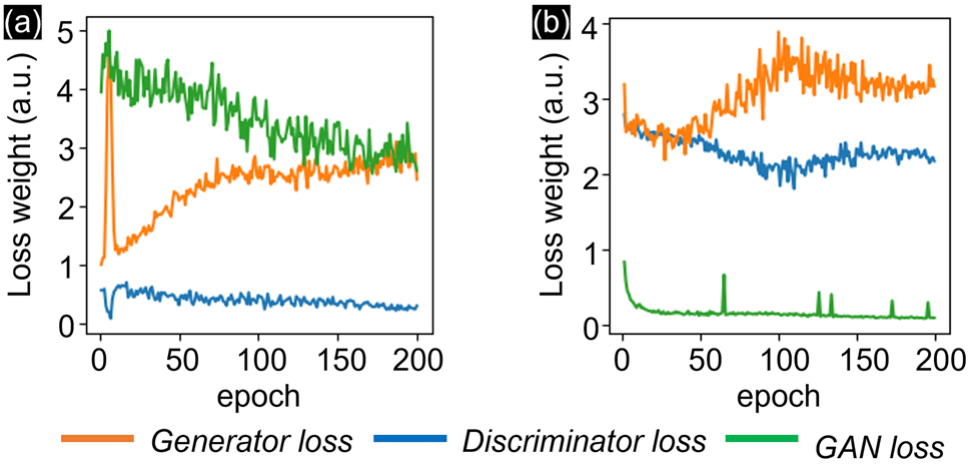
Training regularization of (**a**) cGAN and (**b**) CycleGAN by epochs.

The proposed GANs were implemented in Python 3.7 using the PyTorch framework^57^. The network training was performed with the CUDA acceleration library on NVIDIA RTX 3090. Figure 7 shows that the loss function used for training both cGAN and CycleGAN is L1 loss. Both networks’ weights assigned for loss are 0.01. For optimizing the network, the Adam optimization algorithm was used with a learning rate scheduler to reduce the learning rate by a factor of 0.5 when the initial learning rate was 0.0002. To obtain 200 iterations, cGAN only required around 6 h, which was less than that of CycleGAN that was trained for around 20 h (Table 1). The CW-SSIM was used to analyze 4000 paired b-scans, and the best value of 0.72 was observed at 200 epochs.

**Table 1:** Performance of network (cGAN and CycleGAN).

	cGAN	CycleGAN
Dataset preparation	4000 paired b-scans	4000 unpaired b-scans
Iterations	200	200
Training time	~ 6 h	~ 20 h
Generate speed	37 images/s	11 images/s
Loss function	L1 loss	L1 loss
Conv filter	64	64
Learning rate	0.0002	0.0002
Input/output channel	1	1
Data type	Unsigned 8-bit	Unsigned 8-bit

### Quality comparison

We evaluated the effectiveness of the model and compared it with the target using quality assessment methods. Apart from perceptual quality, three concept metrics, including SNR, CNR, and SSIM, were used for comparisons^25^. The SNR and CNR were used to evaluate the intrinsic quality of the generated images, and SSIM was used to evaluate the generated signal quality compared to the original. SNR was defined as the logarithm of the ratio of the power of a signal ${P}_{signal}$ to the power of the background noise ${P}_{noise}$ (Eq. 4).

4 $$SNR\left(dB\right)=10lo{g}_{10}\left({P}_{signal}/{P}_{noise}\right)$$

CNR was defined as the logarithm of the ratio of biasing between the signal mean ${\mu }_{signal}$ and background ${\mu }_{background}$ to standard deviation of the background ${\sigma }_{background}$ (Eq. 5).

5 $$CNR\left(dB\right)=10*lo{g}_{10}\left(\frac{\left({\mu }_{signal}-{\mu }_{background}\right)}{{\sigma }_{background}}\right)$$

To assess deblurring performance using blind deconvolution, SSIM was used to evaluate the luminance $l$, contrast information $c$, and structural similarity $s$ between the generated signal $G$ and original b-scan image $O$ (Eq. 6).

6 $$SSIM\left(G,O\right)=\left[l\left(G,O\right)*c\left(G,O\right)*s\left(G,O\right)\right]$$

By measuring the lateral and the axial profiles of PAM system and compare with the deep learning models, we used the hair phantom’s cross-section image to present its “full-width half-peak” measuring (FWHM) at focusing depth position.

### Ethics statement

The authors confirm that all methods are based on relevant guidelines and regulations, and that research has been conducted in accordance with the guidelines of ARRIVE. (https://arriveguidelines.org).

### Supplementary Information

Supplementary Information 1.

Supplementary Video 1.

Supplementary Video 2.

Supplementary Video 3.

Supplementary Video 4.

Supplementary Video 5.

Supplementary Video 6.

Supplementary Video 7.

Supplementary Video 8.

Supplementary Video 9.

Supplementary Video 10.

Supplementary Video 11.

## Data Availability

The datasets used and/or analyzed during the current study available from the corresponding author on reasonable request.

## References

[1] Carson, M. K. Alexander Graham Bell: Giving voice to the world (2007).

[2] Kim JY, Lee C, Park K, Han S, Kim C (2016). High-speed and high-SNR photoacoustic microscopy based on a galvanometer mirror in non-conducting liquid. Sci. Rep..

[3] Lee C, Han S, Jeon MY, Kim C, Kim J (2013). Combined photoacoustic and optical coherence tomography using a single near-infrared supercontinuum laser source. Appl. Opt..

[4] Lee D, Lee C, Kim S, Zhou Q, Kim C (2016). In vivo near infrared virtual intraoperative surgical photoacoustic optical coherence tomography. Sci. Rep..

[5] Park EY, Lee D, Lee C, Kim C (2020). Non-ionizing label-free photoacoustic imaging of bones. IEEE Access.

[6] Lee C, Lee D, Zhou Q, Kim J, Kim C (2015). Real-time near-infrared virtual intraoperative surgical photoacoustic microscopy. Photoacoustics.

[7] Park S, Lee C, Kim J, Kim C (2014). Acoustic resolution photoacoustic microscopy. Biomed. Eng. Lett..

[8] Kim J, Park S, Lee C, Kim JY, Kim C (2016). Organic nanostructures for photoacoustic imaging. ChemNanoMat.

[9] Lee MY, Lee C, Jung HS, Jeon M, Kim KS, Yun SH, Kim C (2016). Biodegradable photonic melanoidin for theranostic applications. ACS Nano.

[10] Lee D, Beack S, Yoo J, Kim SK, Lee C, Kwon W (2018). In vivo photoacoustic imaging of livers using biodegradable hyaluronic acid-conjugated silica nanoparticles. Adv. Funct. Mater.

[11] Lee C, Kwon W, Beack S, Lee D, Park Y, Kim H, Hahn SK, Rhee SW, Kim C (2016). Biodegradable nitrogen-doped carbon nanodots for non-invasive photoacoustic imaging and photothermal therapy. Theranostics.

[12] Park B, Lee KM, Park S, Yun M, Choi HJ, Kim J, Lee C, Kim C (2020). Deep tissue photoacoustic imaging of nickel(II) dithiolene-containing polymeric nanoparticles in the second near-infrared window. Theranostics.

[13] Yoo SW, Jung D, Min JJ, Kim H, Lee C (2018). Biodegradable contrast agents for photoacoustic imaging. Appl. Sci..

[14] Lee C, Jeon M, Jeon MY, Kim J, Kim C (2014). In vitro photoacoustic measurement of hemoglobin oxygen saturation using a single pulsed broadband supercontinuum laser source. Appl. Opt..

[15] Lee C, Kim J, Zhang Y, Jeon M, Liu C, Song L, Lovell JF, Kim C (2015). Dual-color photoacoustic lymph node imaging using nanoformulated naphthalocyanines. Biomaterials.

[16] Valluru KS, Willmann JK (2016). Clinical photoacoustic imaging of cancer. Ultrasonography.

[17] Valluru KS, Wilson KE, Willmann JK (2016). Photoacoustic imaging in oncology: Translational preclinical and early clinical experience. Radiology.

[18] Attia ABE, Balasundaram G, Moonthanchery M, Dinish US, Bi R, Ntziachristos V, Olivo M (2019). A review of clinical photoacoustic imaging: Current and future trends. Photoacoustics.

[19] Zhou HC (2019). Optical-resolution photoacoustic microscopy for monitoring vascular normalization during anti-angiogenic therapy. Photoacoustics.

[20] Liu C, Liang Y, Wang L (2019). Optical-resolution photoacoustic microscopy of oxygen saturation with nonlinear compensation. Biomed. Opt. Express.

[21] Guezzi N (2022). Multistage adaptive noise reduction technique for optical resolution photoacoustic microscopy. J. Biophoton..

[22] Tang, J., Xi, L., Jiang, H. Dual focused photoacoustic microscopy. *Biomed. Opt.* (2014).

[23] Vienneau E, Liu W, Yao J (2018). Dual-view acoustic-resolution photoacoustic microscopy with enhanced resolution isotropy. Opt. Lett..

[24] Mozaffarzadeh M (2019). Enhanced contrast acoustic-resolution photoacoustic microscopy using double-stage delay-multiply-and-sum beamformer for vasculature imaging. J. Biophoton..

[25] Guney G (2019). Comparison of noise reduction methods in photoacoustic microscopy. Comput. Biol. Med..

[26] Sun, M., Feng, N., Shen, Y., Shen, X. & Li, J. Photoacoustic signals denoising based on empirical mode decomposition and energy-window method. *Advances in Adaptive Data Analysis* (2012).

[27] Abbasi H, Mostafavi SM, Kavehvash Z (2021). Fast wavelet-based photoacoustic microscopy. JOSA A.

[28] Xing W, Wang L, Maslov K, Wang LV (2013). Integrated optical- and acoustic-resolution photoacoustic microscopy based on an optical fiber bundle. Opt. Lett..

[29] Rajendran P, Sharma A, Pramanik M (2021). Photoacoustic imaging aided with deep learning: A review. Biomed. Eng. Lett..

[30] Gröhl J, Schellenberg M, Dreher K, Maier-Hein L (2021). Deep learning for biomedical photoacoustic imaging: A review. Photoacoustics.

[31] Yang, C., Lan, H., Gao, F. & Gao, F. Deep learning for photoacoustic imaging: A survey. *arXiv* arXiv:2008.04221 (2020).

[32] Hariri A, Alipour K, Mantri Y, Schulze JP, Jokerst JV (2020). Deep learning improves contrast in low-fluence photoacoustic imaging. Biomed. Opt. Exp..

[33] Zhao H (2021). Deep learning enables superior photoacoustic imaging at ultralow laser dosages. Adv. Sci..

[34] Dispirito A (2021). Reconstructing undersampled photoacoustic microscopy images using deep learning. IEEE Trans. Med. Imaging.

[35] Vu T, Li M, Humayun H, Zhou Y, Yao J (2020). Feature article: A generative adversarial network for artifact removal in photoacoustic computed tomography with a linear-array transducer. Exp. Biol. Med..

[36] Guan S, Khan AA, Sikdar S, Chitnis PV (2020). Fully dense UNet for 2-D sparse photoacoustic tomography artifact removal. IEEE J. Biomed. Health Inf..

[37] Sharma A, Pramanik M (2020). Convolutional neural network for resolution enhancement and noise reduction in acoustic resolution photoacoustic microscopy. Biomed. Opt. Exp..

[38] Manwar R (2020). Deep learning protocol for improved photoacoustic brain imaging. J. Biophoton..

[39] Zhao H (2020). A new deep learning method for image deblurring in optical microscopic systems. J. Biophoton..

[40] Kim J (2022). Deep learning acceleration of multiscale superresolution localization photoacoustic imaging. Light Sci. Appl..

[41] Zhang Z (2022). Deep and domain transfer learning aided photoacoustic microscopy: Acoustic resolution to optical resolution. IEEE Trans. Med. Imaging.

[42] Zhang Z (2022). Adaptive enhancement of acoustic resolution photoacoustic microscopy imaging via deep CNN prior. Photoacoustics.

[43] Isola, P., Zhu, J. Y., Zhou, T. & Efros, A. A. Image-to-image translation with conditional adversarial networks. In *Proceedings - 30th IEEE Conference on Computer Vision and Pattern Recognition, CVPR 2017*, 5967–5976 (2017).

[44] Goodfellow I (2020). Generative adversarial networks. Commun. ACM.

[45] Saxena, S. & Teli, M. N. Comparison and Analysis of Image-to-Image Generative Adversarial Networks: A Survey. *arXiv* arXiv:2112.12625 (2022).

[46] Yi X, Walia E, Babyn P (2020). Generative adversarial network in medical imaging: A review. Med. Image. Anal..

[47] Cheng S (2022). High-resolution photoacoustic microscopy with deep penetration through learning. Photoacoustics.

[48] Zhou Y, Sun N, Hu S (2020). Deep learning-powered bessel-beam multi-parametric photoacoustic microscopy. IEEE Trans. Med. Imaging.

[49] Chen AI (2016). Multilayered tissue mimicking skin and vessel phantoms with tunable mechanical, optical, and acoustic properties. Med. Phys..

[50] Cho S, Baik J, Managuli R, Kim C (2020). 3D PHOVIS: 3D photoacoustic visualization studio. Photoacoustics.

[51] da Vinci 1.0 Pro|3D Printers | XYZprinting. https://www.xyzprinting.com/en-US/product/da-vinci-pro.

[52] BD BACTOTM Agar 454g - 214010 | BD. https://www.bd.com/en-us/products-and-solutions/products/product-page.214010#product-tabs-item-d335c68327-tab.

[53] Anesthesia (Guideline) | Vertebrate Animal Research. https://animal.research.uiowa.edu/iacuc-guidelines-anesthesia.

[54] Creswell A (2018). Generative adversarial networks: An overview. IEEE Signal Process. Mag..

[55] Isola, P., Zhu, J. Y., Zhou, T. & Efros, A. A. Image-to-image translation with conditional adversarial networks. In *Proceedings - 30th IEEE Conference on Computer Vision and Pattern Recognition, CVPR 2017*, 5967–5976 (2017).

[56] Zhu, J. Y., Park, T., Isola, P. & Efros, A. A. Unpaired Image-to-Image Translation Using Cycle-Consistent Adversarial Networks. In *Proceedings of the IEEE International Conference on Computer Vision 2017*, 2242–2251 (2017).

[57] Vasilev, I. Python deep learning: exploring deep learning techniques and neural network architectures with PyTorch, Keras, and TensorFlow (2019).

