# Supplementary material for: Enhanced resolution and sensitivity acoustic-resolution photoacoustic microscopy with semi/unsupervised GANs
Source: Sci Rep. 2023 Aug 17;13:13423. doi: 10.1038/s41598-023-40583-x (PMC10435476; doi:10.1038/s41598-023-40583-x)
Supplement: Supplementary file 1 — Supplementary Information 1. [file 41598_2023_40583_MOESM1_ESM.docx]

**Supplementary materials**

**Enhanced resolution and sensitivity acoustic-resolution photoacoustic microscopy with semi/unsupervised GANs**

**Thanh Dat Le^1^, Jung-Joon Min^2^, and Changho Lee^1,2*^**

^1^Department of Artificial Intelligence Convergence, Chonnam National University, Gwangju 61186, Korea.

^2^Department of Nuclear Medicine, Chonnam National University Medical School & Hwasun Hospital, 264, Seoyang-ro, Hwasun-eup, Hwasun-gun, Jeollanam-do, 58128, Korea.

^*^ Correspondence: ch31037@jnu.ac.kr; Tel.: +82-61-379-2885


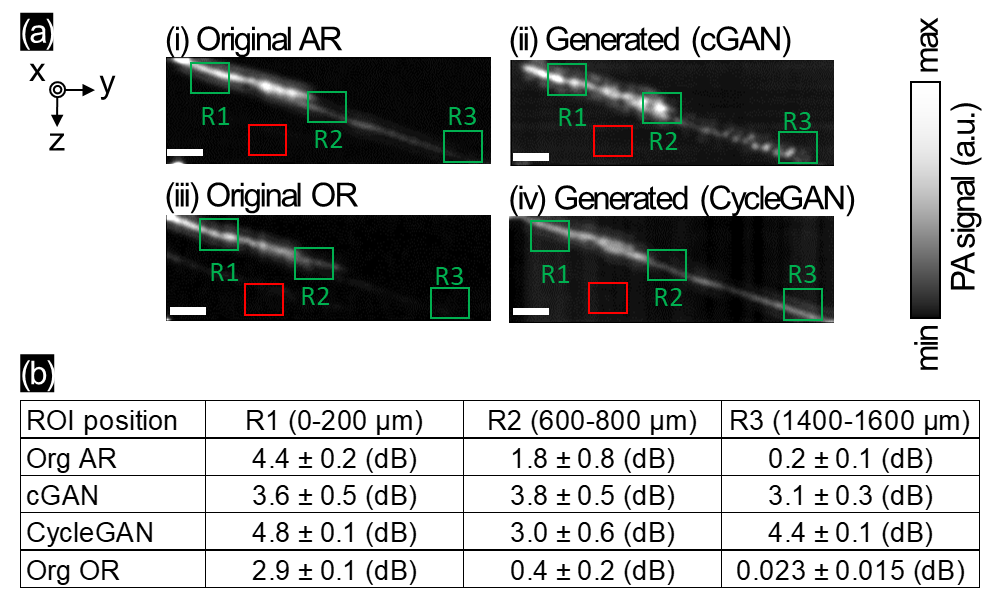


**Supplementary Fig. S1**: (a) The SNR measurement at different three depth at Fig. 1(d), (b) The measured SNR table.


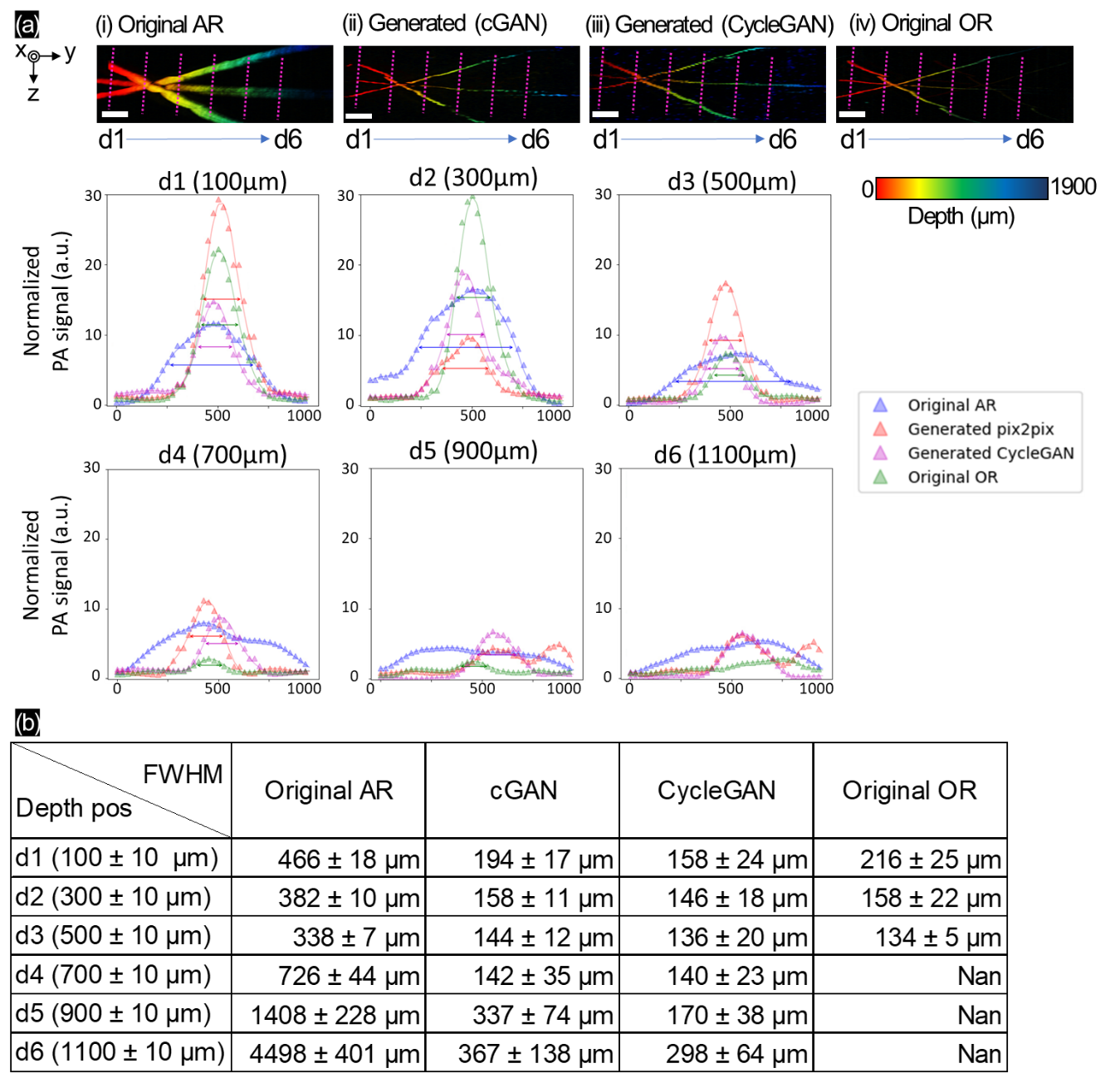


**Supplementary Fig. S2**: (a) Measuring FWHM of lateral profiles by depth changing from d1 to d6 (100 μm to 1100 μm) at red-dotted lines of Fig. 1(c), (b) The measured FWHMs table at position 100µm to 1100µm.


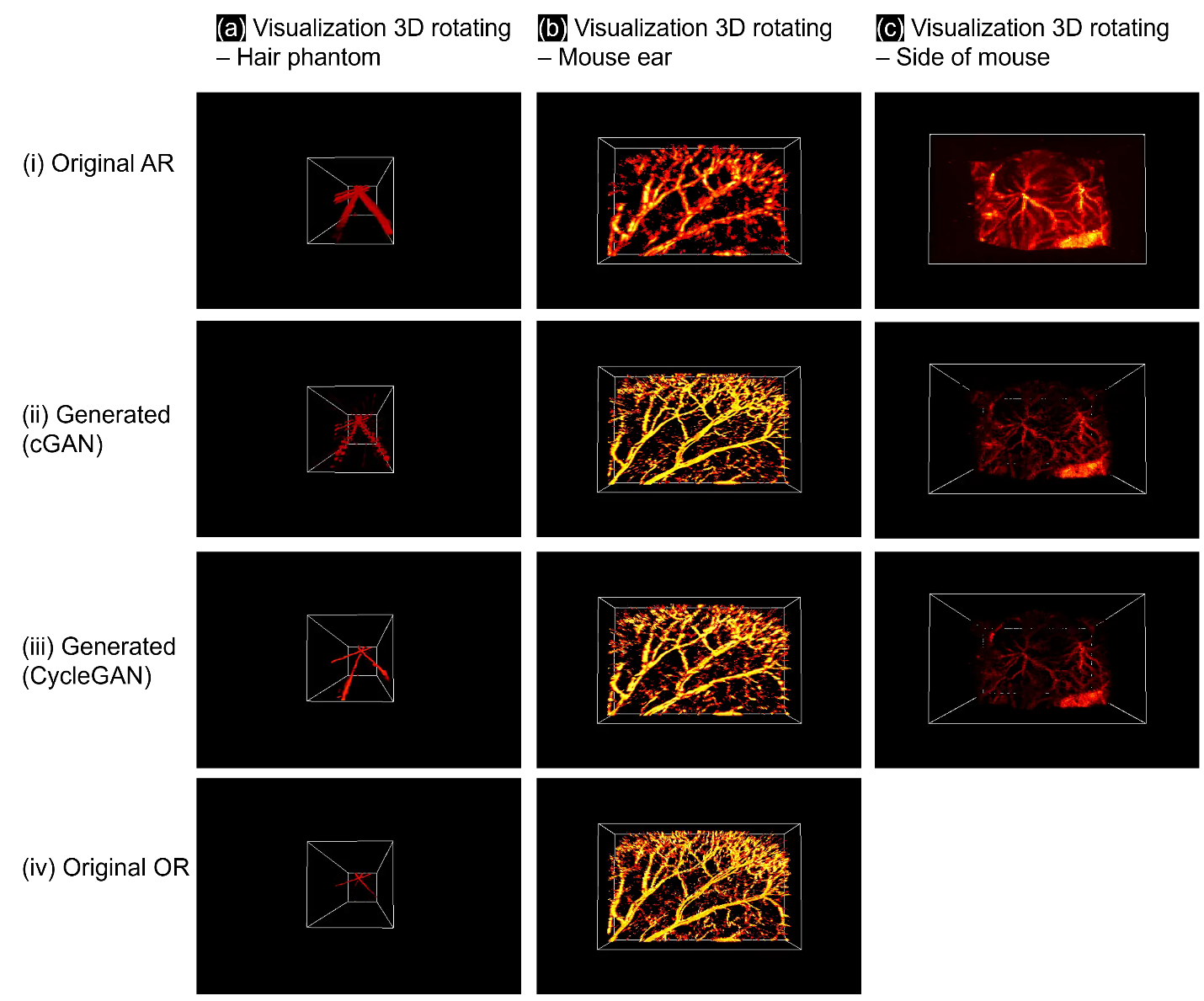


**Supplementary Fig. S3.** Visualization 3D images movies (a) hair phantom (Movie 1), (b) Mouse ear (Movie 2), and (c) Mouse skin (Movie 3) (x-axis: red, y-axis: green, z-depth: blue) showed in (i) Original AR, (ii) Generated by cGAN, (iii) Generated by CycleGAN. (iv) Original OR.


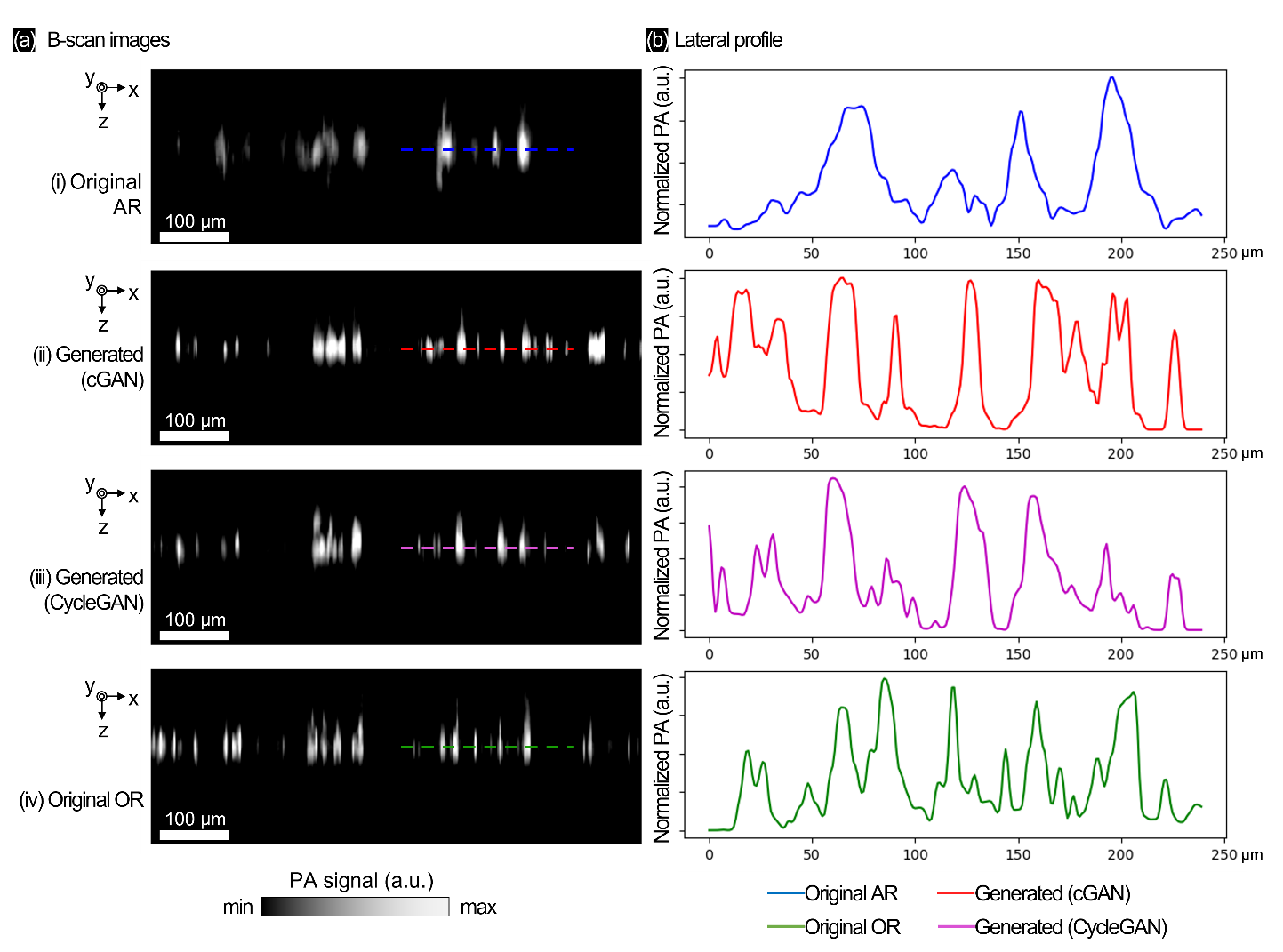


**Supplementary Fig. S4.** The b-scan profile at yellow line in mouse ear of Fig. 2(a) B-scan images and (b) its lateral profile showed in (i) Original AR, (ii) Generated by cGAN,(iii) Generated by CycleGAN, (iv) Original OR.


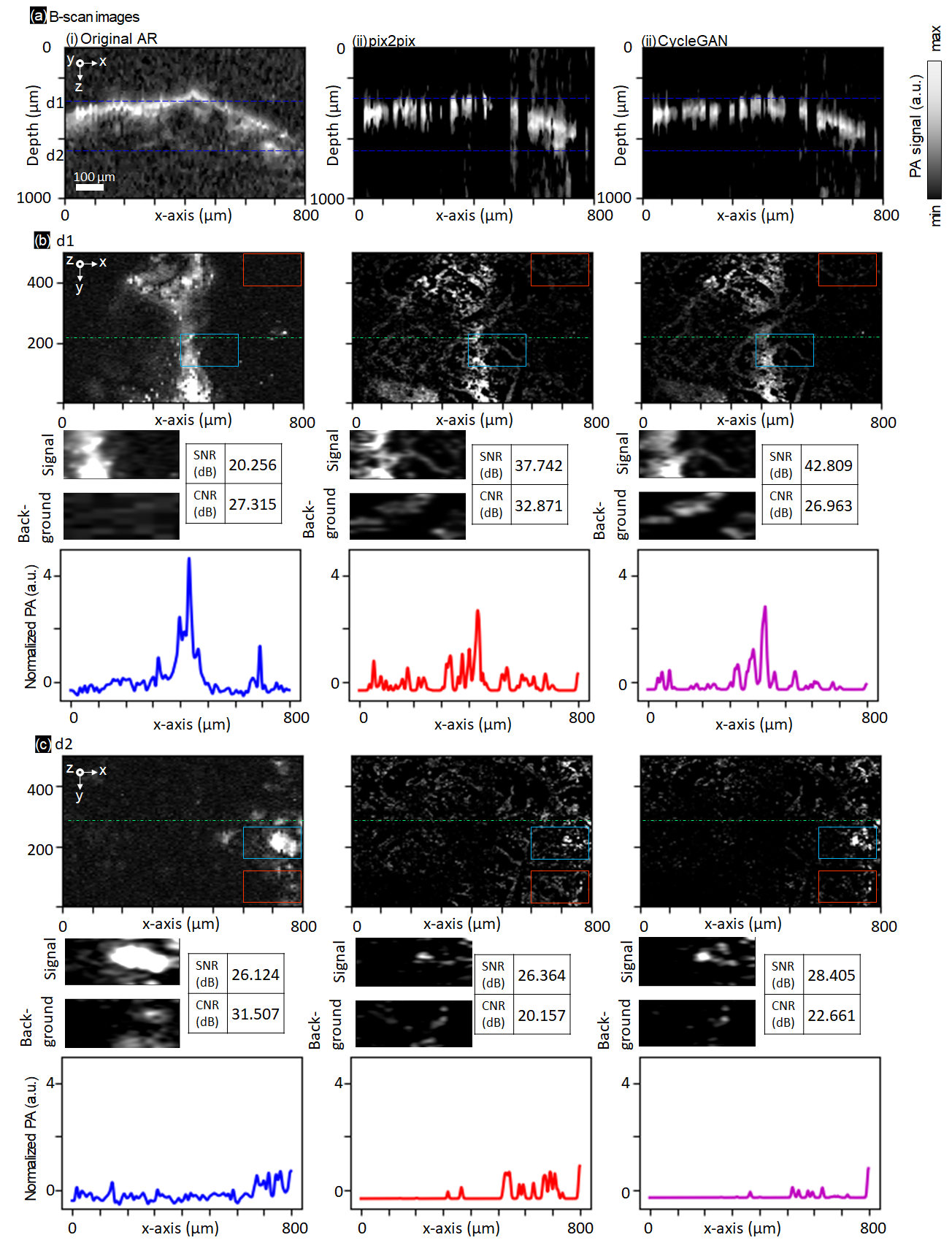


**Supplementary Fig. S5.:** (a) Cross-sectional images at at yellow line in Fig. 3 (a), (b) MAP images, SNRs & CNRs, and corresponding lateral profiles at two different depth (d1 and d2) of cross-section images.
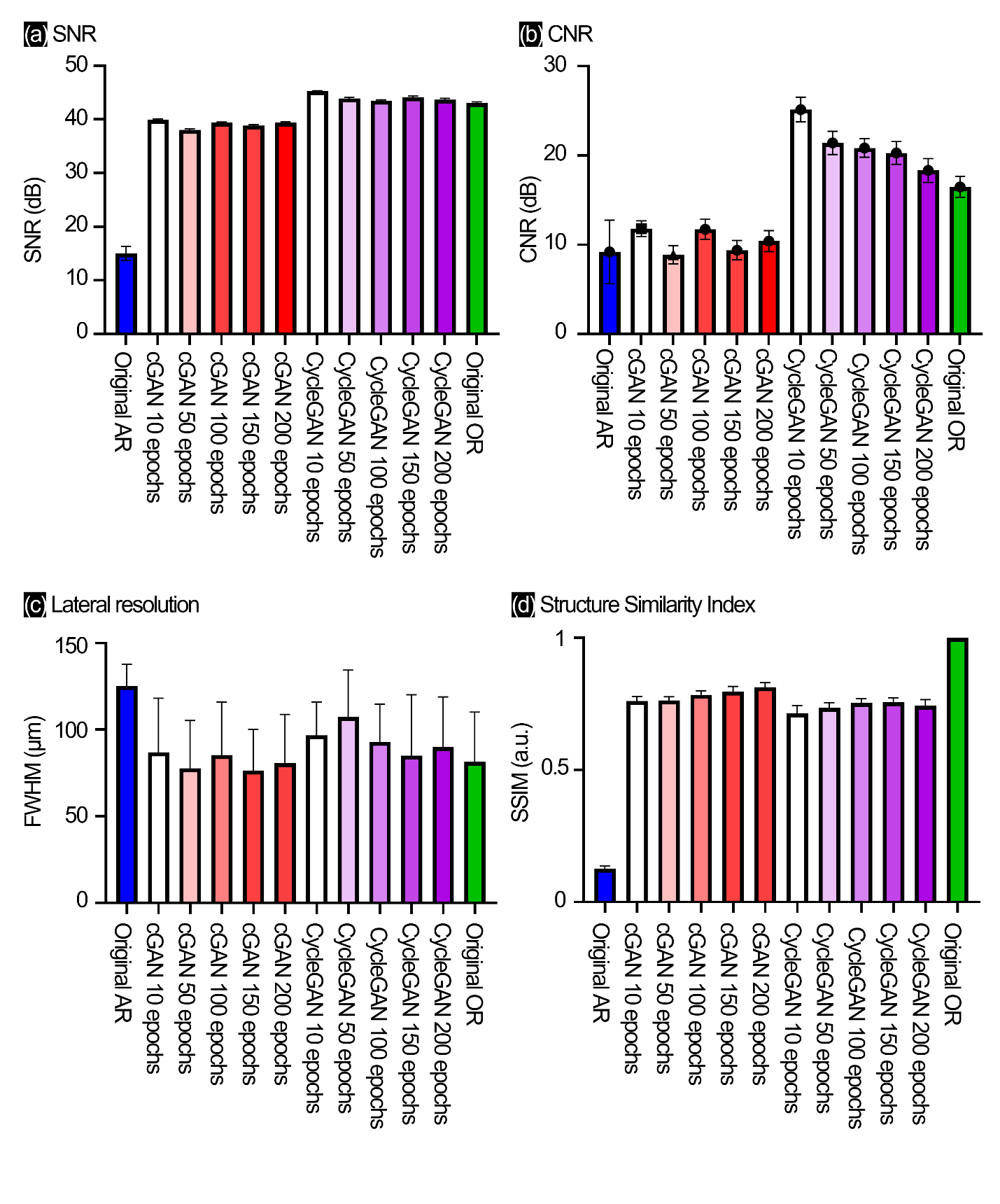


**Supplementary Fig. S6.** Quality measurement during training process with cGAN and CycleGAN by 10-200 epochs. The measured (a) SNR, (b) CNR, (c) Lateral resolution, and (d) Similarity index SSIM.
